# Single-agent versus combination regimens containing propofol: a retrospective cohort study of recovery metrics and complication rates in a hospital-based endoscopy suite

**DOI:** 10.1016/j.bjane.2025.844602

**Published:** 2025-02-27

**Authors:** Guozhen Xie, Maria Estevez, Kiyan Heybati, Matthew Vogt, Michael Smith, Christine Moshe, Johanna Chan, Vivek Kumbhari, Ryan Chadha

**Affiliations:** a Mayo Clinic Alix School of Medicine, Mayo Clinic, Rochester, USA; b Clinical Studies Unit, Mayo Clinic Jacksonville, FL, USA; c Department of Anesthesiology and Perioperative Medicine, Mayo Clinic, Rochester, MN, USA; d Department of Anesthesiology and Perioperative Medicine, Mayo Clinic Jacksonville, FL, USA; e Department of Gastroenterology and Hepatology, Mayo Clinic Jacksonville, FL, USA

**Keywords:** Adverse events, Anesthesia, Endoscopy, Propofol

## Abstract

**Background:**

Anesthesiologists are often tasked with overseeing sedation in non-surgical settings. We aim to determine whether adding adjuvant sedatives to propofol affects the recovery times and complication rates after endoscopy.

**Methods:**

We conducted a retrospective cohort study of adults (≥18) who received propofol while undergoing esophagogastroduodenoscopy (EGD) and/or colonoscopy (COL) at a large academic institution over a four-year period. Patients receiving propofol alone were compared against patients receiving propofol in combination with midazolam, fentanyl, ketamine, or dexmedetomidine. The primary outcome was PACU length of stay, adjusted for age, sex, and ASA Score. Secondary outcomes included incidence of PACU postoperative nausea and vomiting, hypoxemia (SpO_2_ < 90%), bradycardia (HR < 60 bpm), and escalation of care (hospital admission), reported in adjusted odds ratios and their 95% confidence intervals.

**Results:**

Across the study period, 28,532 cases were included. Colonoscopies performed under propofol+fentanyl sedation were associated with significantly longer PACU LOS compared to propofol alone. Adjusted mean PACU LOS was significantly longer in patients receiving adjuvant fentanyl, compared to propofol alone (p < 0.01) and propofol + dexmedetomidine (p < 0.01). Patients receiving propofol alone exhibited a 9.4% incidence of bradycardia, 16.0% hypoxia, 0.89% PONV, and 0.40% hospitalization. Adjuvant fentanyl use was associated with higher odds of hypoxia across all procedure types (p < 0.05). Adjuvant dexmedetomidine was associated with higher rates of bradycardia, but lower rates of hypoxia, PONV, and hospitalization (p < 0.05).

**Conclusions:**

With the exception of fentanyl, combining propofol with other sedatives was not associated with longer recovery times. The incidence of complications differed significantly with the use of adjuvant fentanyl or dexmedetomidine.

## Introduction

The number of endoscopic procedures performed annually in the US continues to increase,^1^ and various regimens are used for sedation in the procedural setting. Recent decades have seen a rise in anesthesia-supported sedation, which leverages the anesthesiologist's expertise in sedatives and endotracheal intubation to improve patient comfort and safety.

Owing to its reliability and rapid onset of action, propofol is among the most popular sedative agents. Large observational studies (n > 100,000) have reported on the extremely low complication rates associated with propofol sedation,^2^, ^3^, ^4^ and randomized controlled studies have attempted to compare single-agent versus combination regimens containing propofol in regards to recovery times,^5^, ^6^, ^7^ patient satisfaction scores,^8^, ^9^, ^10^, ^11^ and complication rates.^3^^,^^4^^,^^12^, ^13^, ^14^, ^15^, ^16^, ^17^, ^18^ Unfortunately, the size and direction of the effects vary by report. For example, Molina-Infante et al. reported that combining propofol with midazolam prolonged recovery times after colonoscopy,^7^ whereas Julian-Gomez et al. reported no difference.^19^ Pooled analysis is difficult due to the various procedures, sedatives, and outcomes represented in these studies. The ASA's meta-analyses of propofol versus *single-agent* alternatives have shown decreased recovery time and recall in the propofol groups but equivocal rates of hypoxia.^20^ No such analysis has been conducted for combination therapies involving propofol. While dexmedetomidine has been studied as an adjuvant or alternative to benzodiazepines or opioids, combination therapy with propofol is not discussed. The ambiguity in the existing literature is reflected in the ASA guidelines on moderate procedural sedation, which broadly calls for clinical judgment on a case-by-case basis.^20^

Due to propofol's relatively good safety profile, the complication rates associated with propofol sedation are low and difficult to quantify. Even when an adequate sample size is achieved, as in the ProSed2 study,^4^ medication selection and post-procedural monitoring are often not standardized across participating centers, leaving room for confounding effects. It follows that a single-center study may allow for a higher degree of consistency and standardization, but to our knowledge, no single institution has performed a study of comparable size/power. Our institution performs over 10,000 endoscopy procedures annually, half of which are performed under anesthesia-supported sedation with propofol. Hospital policy states that only anesthesiologists, anesthesiologists in training, and nurse anesthetists are permitted to administer propofol. Furthermore, all nurse anesthetists and trainees must work under supervision of an anesthesiologist.

This retrospective cohort study represents the largest single-center study to date comparing single-agent propofol to combination regimens, and it is among the first to characterize recovery times, hospitalization rates, and postoperative nausea and vomiting (PONV) simultaneously in a non-surgical setting.^21^ We hypothesized that propofol with adjuvants is associated with longer recovery times and increased rates of complications compared to propofol alone.

## Methods

This study was conducted in accordance with the guidelines for Strengthening the Reporting of Observational Studies in Epidemiology (STROBE) (ESM 1).

### Study design

We conducted a retrospective cohort study including adults (≥ 18) undergoing EGD and/or colonoscopy under propofol sedation at our institution between October 1, 2018, through December 31, 2022. Data were retrieved from institutional data warehouses that provide a near real-time replica of our hospital's Electronic Healthcare Record (EHR).

### Data filtering and cohort construction

The original 63,663 records contained 180 unique procedure codes, including “Colonoscopy”, “Colonoscopy [with] possible biopsy”, “Colonoscopy [with] endoscopic mucosal resection”, among others. Based on independent review by two investigators, equivalent or comparable procedures were reclassified as “EGD”, “Colonoscopy”, or “Both” and all other procedures were excluded. Data was further filtered for completeness, and any entries with missing demographic or outcome data (with the exception of missing race and ASA Rating) were excluded.

For each patient, sedation type was determined by filtering the dataset based on drugs infused. Patients not receiving propofol were excluded, as well as patients who were electively or emergently intubated. All patients receiving propofol with rocuronium or succinylcholine were excluded as “General Anesthesia”, regardless of other infusions. Of the remaining patients, those receiving propofol were further categorized as “Prop” for propofol, “Prop+Benzo” for adjuvant midazolam, “Prop+Fent” for adjuvant fentanyl, “Prop+Ket” for adjuvant ketamine, “Prop+Dex” for adjuvant dexmedetomidine, and “Prop+Multi” for multiple simultaneous adjuvants.

### Outcomes

The primary outcome was PACU length of stay ‘PACU LOS’, which was calculated as the difference between Procedure End Time and Recovery Phase II End Time and reported in minutes. Date and time parameters were formatted as ‘MM/DD/YYYY hh:mm’ by default, and calculations were performed using Excel and verified using the dplyr:lubridate package in R.

Secondary outcomes included postoperative nausea and vomiting (PONV), hypoxemia (SpO_2_ < 90%), bradycardia (HR < 60 bpm), and escalation of care (hospital admission). Complications were identified using a combination of procedure records, vital signs data, and ICD10 diagnoses entered in the PACU. Absolute incidence and percentages were reported, and relative incidence was expressed as an odds ratio between adjuvant and propofol.

### Statistical analysis

All statistical analysis was conducted in R4.1.3 in accordance with CRAN policies on open-source use.

The effect of adjuvant medications on PACU LOS was modelled using a multivariate linear model to adjust for Age, Sex, procedure type, and ASA Score. Adjusted mean PACU LOS was calculated using the emmeans package using median values as a reference point (ASA 3, age 63, female sex), and pairwise comparisons were conducted using the Scheffe Test.

The effects of adjuvant medications on complication rates were modelled using logistic regression while adjusting for age, sex, procedure type, and ASA score. Adjusted odds ratios and 95% confidence intervals were computed by applying the Wald Test. Unadjusted incidence counts and rates were also reported for the sake of transparency.

## Results

### Patient demographics

63,663 procedures were performed between October 1, 2018, through December 31, 2022. All procedures were screened, and 6,501 were excluded based on procedure type, 28,375 were excluded based on sedatives used, and 255 were excluded for incomplete data, leaving 28,532 procedures for analysis. Of the included procedures, 54% were performed on females and 88% on Caucasians. Average patient age was 58 (SD = 14.4), median age was 61 (IQR 49‒70), and average ASA score was 2.57. Baseline demographics were broadly consistent across categories, with a few notable exceptions (Table 1). 16,206 patients (56% of total included) were documented ASA score 3 or higher.

**Table 1:** Demographic information and multilinear regression for analysis of covariates of PACU LOS.

Sedation	% Female	% Caucasian	Mean Age (SD)	Median Age	Mean ASA
Prop	55.08	88.01	59.81 (15.2)	62.0	2.58
Prop+Fent	50.78	88.74	54.05 (16.6)	57.0	2.54
Prop+Benzo	67.82	89.94	46.61 (16.0)	47.0	2.45
Prop+Ket	50.77	84.62	47.20 (17.7)	48.0	2.80
Prop+Dex	55.16	87.44	50.90 (16.6)	52.0	2.52
Prop+Multi	50.00	86.70	44.59 (16.5)	44.5	2.55

### Sedative agents

Of the included patients, 23,607 received prop, 2,054 received prop+dex, 2,052 received prop+fent, 406 received prop+multi, 348 received prop+benzo, and 65 received prop+ket. Fentanyl was the most common adjuvant in colonoscopies, and dexmedetomidine was the most common adjuvant in EGD and combined procedures (Table 2).

**Table 2:** Mean and median PACU LOS (minutes) with associated intervals.

Procedure	Adjuvant Type	N	Mean LOS	Adjusted Mean LOS	95% CI of mean (lower)	95% CI of mean (upper)	Median LOS	25^th^ % tile	75^th^ % tile
EGD	Prop	9622	31.71	33.05	32.17	33.93	26.0	17.00	43.00
	Prop+Fent	1052	35.07	36.77	34.99	38.56	27.0	17.00	44.00
	Prop+Benzo	128	33.12	38.28	34.28	42.29	25.5	18.00	41.00
	Prop+Ket	48	29.08	30.32	21.24	39.40	26.0	16.00	38.25
	Prop+Dex	901	30.11	31.53	29.74	33.32	25.0	19.00	38.00
	Prop+Multi	180	36.20	35.94	32.20	39.69	27.0	19.00	43.25
Colonoscopy	Prop	9008	32.63	34.48	33.49	35.46	28.0	17.00	43.00
	Prop+Fent	371	39.95	38.20	36.27	40.12	33.0	21.00	48.00
	Prop+Benzo	115	45.78	39.71	35.68	43.74	29.0	20.00	45.50
	Prop+Ket	6	29.33	31.74	22.62	40.86	28.0	23.25	36.50
	Prop+Dex	539	31.34	32.95	31.06	34.84	27.0	19.00	41.00
	Prop+Multi	86	35.56	37.37	33.57	41.17	30.0	21.25	38.75
Combined	Prop	4977	33.31	34.38	33.27	35.48	27.0	18.00	44.00
	Prop+Fent	629	34.82	38.10	36.21	39.99	29.0	19.00	45.00
	Prop+Benzo	105	36.09	39.61	35.57	43.65	30.0	20.00	46.00
	Prop+Ket	11	30.64	31.65	22.53	40.77	24.0	20.00	41.00
	Prop+Dex	614	31.81	32.86	30.97	34.74	28.0	20.00	39.00
	Prop+Multi	140	33.76	37.27	33.49	41.05	30.0	20.75	45.00

### Length of stay

With the exception of prop+fent, adjuvant groups did not differ significantly in mean PACU LOS. Among patients receiving propofol alone, adjusted mean PACU LOS was 33.05, 34.48, 34.38 minutes following EGD, COL, and Combined procedures, respectively. This was compared against 36.77, 38.20, 38.10 for prop+fent, 38.28, 39.71, 39.61, for prop+benzo; 30.32, 31.74, 31.65 for prop+ket, 31.53, 32.95, 32.86 for prop+dex; and 35.94, 37.37, 37.27 for prop+multi.

**Figure 1:**
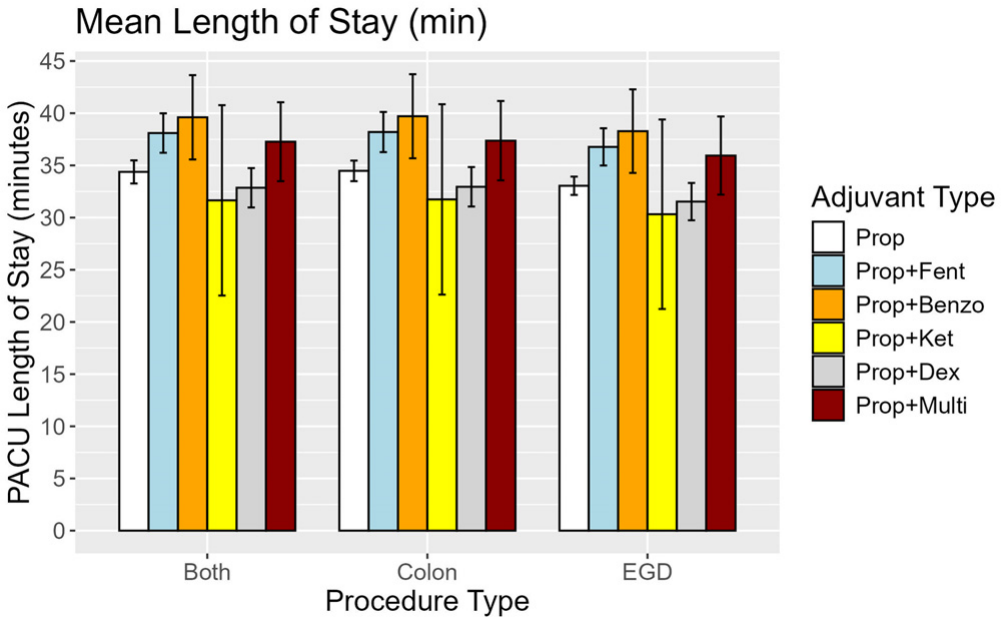
Adjusted mean PACU LOS with associated 95% Confidence Intervals.

**Figure 2:**
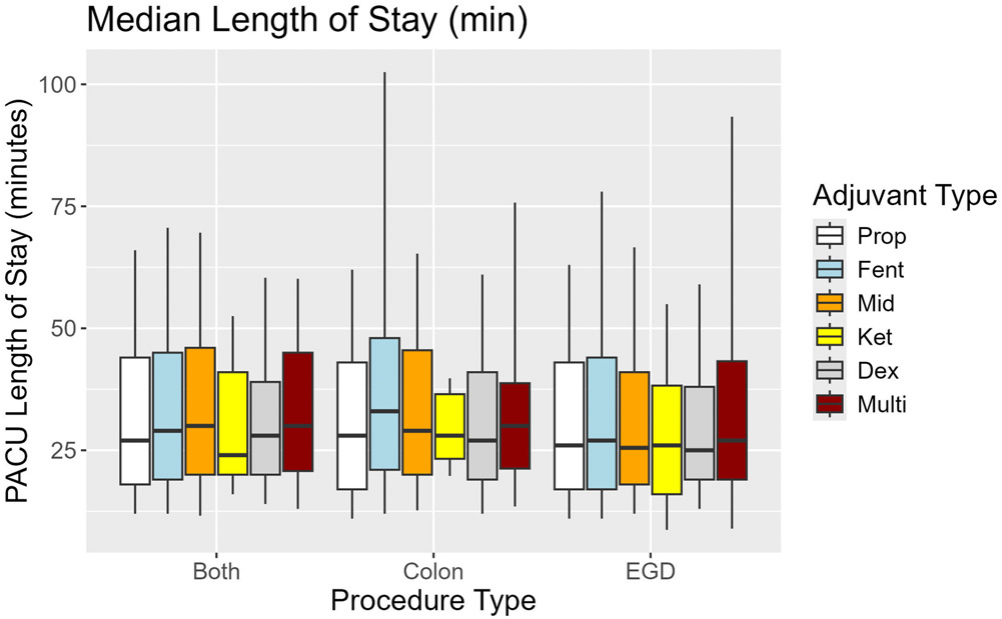
Unadjusted Median PACU LOS with associated IQR and 5^th^, 95^th^ percentile ranges.

Adjusted PACU LOS differed significantly between prop+fent and other groups, specifically prop (p < 0.01) and prop+dex (p < 0.01). Among prop+fent patients, median PACU LOS was 27, 33, and 29 for EGD, COL, and Combined procedures, respectively (Table 2, Fig. 1, 2). No significant differences in median PACU LOS were observed between prop and any other group, specifically prop+dex, prop+benzo, prop+ket, or prop+multi.

### Complication rates

**Figure 3:**
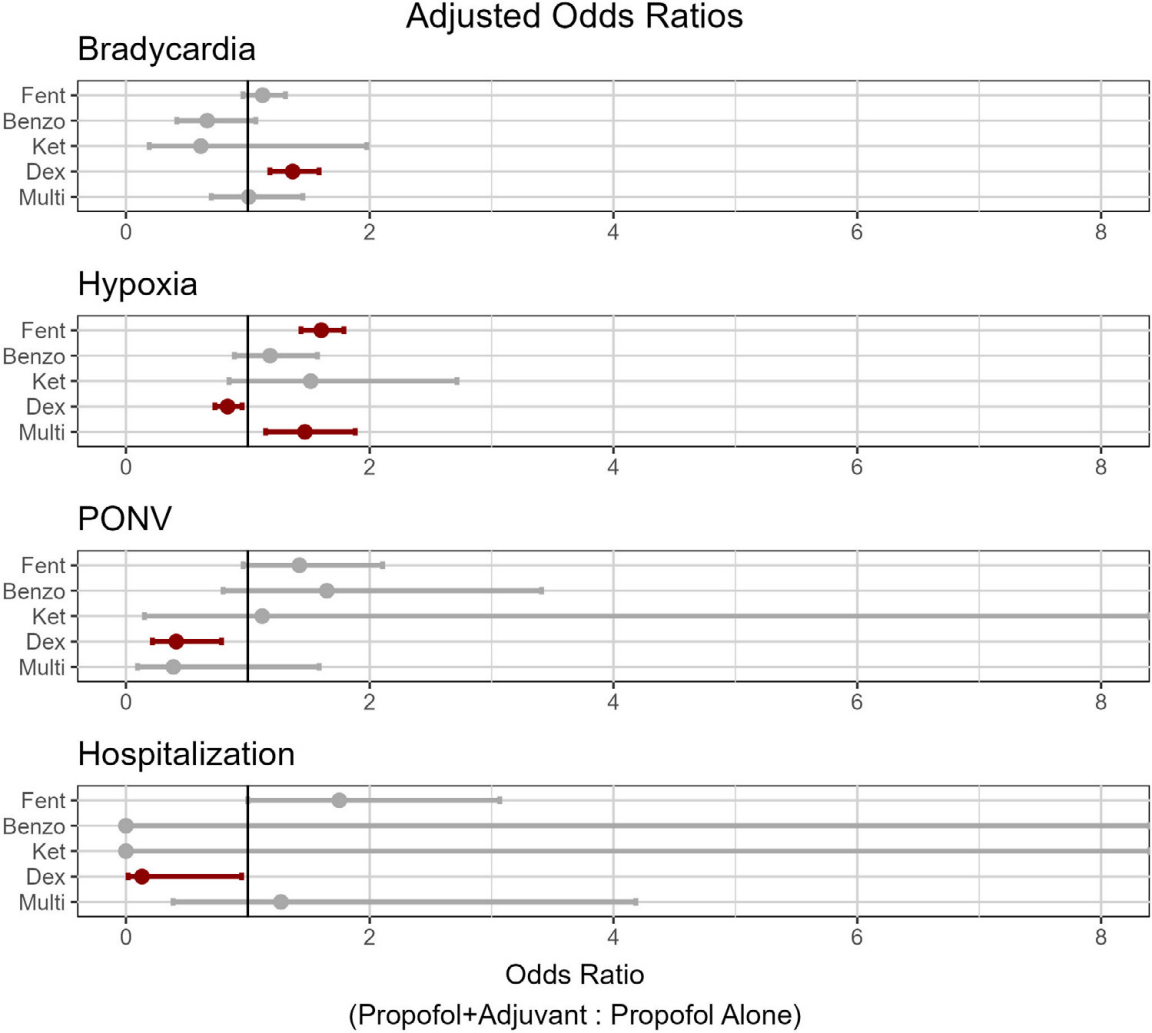
Odds ratios of complications.

Hypoxia was the most common PACU complication across all procedure and sedation types, with an incidence rate of 16.5%, followed by bradycardia at 9.5%, PONV at 0.9%, and hospitalization at 0.4% (Table 3). Compared to prop, prop+fent and prop+multi patients had higher odds of exhibiting hypoxia across all procedure types, whereas prop+dex patients had lower odds (Fig. 3).

**Table 3:** Incidence of PACU complications.

Procedure	Adjuvant Type	Total	Bradycardia (%)	Hypoxia (%)	PONV (%)	Hospitalization (%)
EGD	Prop	9622	735 (7.6)	1776 (18.5)	111 (1.2)	42 (0.4)
	Prop+Fent	1052	77 (7.3)	270 (25.6)	12 (1.1)	13 (1.2)
	Prop+Benzo	128	8 (6.2)	30 (23.4)	3 (2.3)	0 (0.0)
	Prop+Ket	48	1 (2.0)	12 (25.0)	1 (2.0)	0 (0.0)
	Prop+Dex	901	71 (7.8)	130 (14.4)	2 (0.2)	1 (0.1)
	Prop+Multi	180	16 (8.8)	38 (21.1)	0 (0.0)	1 (0.5)
Colonoscopy	Prop	9008	918 (10.2)	1026 (11.4)	44 (0.5)	28 (0.3)
	Prop+Fent	371	51 (13.7)	55 (14.8)	7 (1.8)	0 (0.0)
	Prop+Benzo	115	5 (4.3)	10 (8.7)	0 (0.0)	0 (0.0)
	Prop+Ket	6	0 (0.0)	1 (16.6)	0 (0.0)	0 (0.0)
	Prop+Dex	539	69 (12.8)	48 (8.9)	2 (0.3)	0 (0.0)
	Prop+Multi	86	9 (10.4)	16 (18.6)	0 (0.0)	0 (0.0)
Combined	Prop	4977	572 (11.5)	971 (19.5)	54 (1.1)	24 (0.5)
	Prop+Fent	629	76 (12.0)	165 (26.2)	11 (1.7)	2 (0.3)
	Prop+Benzo	105	6 (5.7)	20 (19.0)	5 (4.7)	0 (0.0)
	Prop+Ket	11	2 (18.1)	2 (18.1)	0 (0.0)	0 (0.0)
	Prop+Dex	614	94 (15.3)	101 (16.4)	6 (0.9)	0 (0.0)
	Prop+Multi	140	8 (5.7)	30 (21.4)	2 (1.4)	2 (1.4)

Prop+dex was associated with higher rates of bradycardia and lower rates of PONV and hospitalization compared to prop. Prop+benzo and prop+ket did not exhibit significant differences compared to prop (Fig. 3). Certain prop+benzo and prop+ket sample sizes were insufficient to assess for complication rates.

## Discussion

In this study, we retrospectively examined the PACU course of over 20,000 patients undergoing endoscopic procedures at a large academic institution. Length of stay has not been studied in the context of sedative regimens containing propofol. In this study, only fentanyl was associated with a sufficient change in PACU LOS that met the predefined thresholds for statistical significance.

Complications and side effects of the anesthetic agents under study are well documented in the literature. We observed a higher incidence of hypoxia among prop+fent patients, which is unsurprising given its known propensity for respiratory depression. We observed decreased rates of hypoxia among prop+dex patients, which is broadly consistent with its applications in the OR and ICU as a non-respiratory depressive sedative agent. Prop+dex patients also exhibited more bradycardia, which is a known adverse effect of the drug, as well as decreased PONV and hospitalization, which are not documented elsewhere in the literature but could be a product of lower propofol dosage to achieve the same level of sedation. Interestingly, while opioids are believed to contribute to PONV,^22^ the odds ratio was not significant when comparing PONV rates between prop+fent and prop groups. The incidence of PONV has not been widely studied in the procedural setting, but other risk factors include usage of nitrous oxide,^23^^,^^24^ inhaled anesthetics,^25^ duration of anesthesia,^22^ and age younger than fifty.^26^ Of these, only duration, age, and perioperative opioid use were relevant to this study. Average age was 5.8-years younger in the prop+fent group, but this was accounted for in our logistic model. Our observed rates of PONV were significantly lower than those reported previously,^27^ possibly due to the lower level of sedation and the lack of aforementioned risk factors. We also attempted to compare hospitalization rates between subgroups, but even with over 200 hospitalizations, the number of cases in each subgroup was often insufficient for analysis and calculation of odds ratios (Fig. 3). Overall complication rates of PONV and hospitalization were extremely low across all sedation types.

We did observe differences in baseline demographics, notably age and sex, and we performed multilinear regression to adjust for confounding effects of age, sex, and ASA score on PACU LOS. We also performed logistic regression to adjust for confounding effects on rates of hypoxia, bradycardia, PONV, and hospitalization. Interestingly, our coefficients imply that age and ASA are weak confounders at best, suggesting that perhaps providers are already successful in tailoring their anesthetic plans and dosages to sicker and older patients. Female sex was associated with a significantly higher PACU LOS, and this effect was corrected by calculating estimated marginal means.

The strengths of this study include a large sample size, uniform recovery protocols, and a wide range of both medications and complications assessed. We believe that at the time of authorship, this study represents the largest single-center study of propofol sedation to date. As previous investigators have observed, a large sample size is needed to measure the incidence of rare complications that often go undetected in smaller samples, but the lack of standardization across multi-center studies presents a challenge to controlled comparison.^4^ In contrast, all patients included in this study were monitored in the same recovery unit, thus ensuring uniform recovery and discharge protocols. Our status as a hospital-based endoscopy suite also allows us to report on hospitalization, which has significant implications for patient comfort and safety, as well as resource utilization.

The weaknesses of this study include the lack of randomization and medication dosage data. As an observational study, our results are subject to confounding factors, including those listed in Table 1, as well as factors not captured in our dataset. Although robust statistical methods were used to account for baseline differences in age, sex, and ASA score, all models will exhibit residuals that are not predicted or accounted for. In addition, confounding variables may exist outside our dataset, to which the only solution would be randomization. Medication dosage represents a potential unmeasured confounder in this study. While dosages are typically adjusted to body weight, differences in dosing may partially account for differences in PACU LOS, as well as any dose-dependent complications of procedural sedation. Finally, generalizability is negatively impacted by the single-center nature of this study. Given these limitations, additional prospective investigation is needed to verify the findings described above.

## Conclusions

The addition of fentanyl may be associated with prolonged recovery, whereas the addition of dexmedetomidine, midazolam, or ketamine does not correlate strongly with recovery times. Fentanyl is associated with higher rates of hypoxia, whereas dexmedetomidine is associated with lower rates of hypoxia, but higher rates of bradycardia. Overall complication rates were low across all sedation types. Taken together, this data suggests that more research is needed in this area, and for now propofol remains a relatively safe and effective sedative agent regardless of adjuvant medications used, although rates of adverse effects should be considered.

## Conflicts of interest

The authors declare no conflicts of interest.
